# Two New Sturgeon Species are Susceptible to Acipenser Iridovirus European (AcIV-E) Infection

**DOI:** 10.3390/pathogens9030156

**Published:** 2020-02-25

**Authors:** Davide Mugetti, Paolo Pastorino, Vasco Menconi, Margherita Messina, Loretta Masoero, Luisa Ceresa, Claudio Pedron, Marino Prearo

**Affiliations:** 1 Istituto Zooprofilattico Sperimentale del Piemonte, Liguria e Valle d’Aosta, 10154 Torino, Italy; vasco.menconi@izsto.it (V.M.); margherita.messina@izsto.it (M.M.); loretta.masoero@izsto.it (L.M.); marino.prearo@izsto.it (M.P.); 2 Dipartimento di Scienze della Vita, Università degli Studi di Trieste, 34127 Trieste, Italy; 3 Dipartimento di Scienze Veterinarie, Università degli Studi di Torino, 10095 Grugliasco (TO), Italy; ceresa.luisa1@gmail.com; 4 Independent Researcher, 20090 Settala (MI), Italy; claudio.pedron@alice.it

**Keywords:** Viral diseases, nucleocytoplasmic large DNA viruses, sturgeon, *Acipenser stellatus*, *Acipenser ruthenus*

## Abstract

We report the first case of Acipenser iridovirus European (AcIV-E) infection in starry sturgeon (*Acipenser stellatus*) and in sterlet (*A. ruthenus*) reared in Northern Italy. During 2018, mortality began in *A. stellatus* and *A. ruthenus* specimens reared in co-habitation with Russian sturgeon positive for AcIV-E. Molecular analyses were done on the gills to amplify a fragment of the major capsid protein (MCP) gene using real-time PCR against AcIV-E. DNA of the positive samples was further sequenced and phylogenetic analyses were performed. The MCP gene sequences were highly similar to a virus previously identified in Italy (nucleotide identities between 99.38% and 99.69%). Phylogenetic analysis confirmed our hypothesis of passage of the virus from the infected Russian sturgeon. The detection of AcIV-E in new species of the Acipenseridae family may impact on sturgeon production, with relevant economic losses.

## 1. Introduction

Acipenser Iridovirus European (AcIV-E) is an emerging pathogen in European sturgeon farming [1,2]. AcIV-E and other related viruses affecting sturgeon, including White Sturgeon Iridovirus (WSIV) and Namao virus (NV), were initially classified as *Iridoviridae*, but no specific genus could be assigned [3]. Although fragmented, available genomic data allowed the classification of these viruses as Nucleocytoplasmic Large DNA Viruses (NCLDVs) of the order Megavirales [4,5]. The order also includes viruses of the Mimiviridae family, which are closely related to pathogenic sturgeon viruses [4]. These taxonomic data, combined with the presence of two different variants of AcIV-E, require a revision of the current nomenclature, as advocated by other authors [6]. 

The first reports of AcIV-E date between 2012 and 2015 in studies conducted in three western European countries involving four species: Russian sturgeon (*Acipenser gueldenstaedtii*), Siberian sturgeon (*A. baerii*), Adriatic sturgeon (*A. naccarii*), and Beluga (*Huso huso*) [1,2]. These cases were followed by a description of a mortality event in young Siberian sturgeon individuals imported into Sweden [7]. Nevertheless, literature on AcIV-E in Russian sturgeon is scant [5,6]. 

Russian sturgeon seems to be the species most susceptible to AcIV-E infection [5,8], though mortality has been reported in other sturgeon species (*A. baerii*, *A. naccarii*, *H. huso*) [1,2,7]. It is important to obtain information about AcIV-E infection and transmission in other species of the Acipenseridae family since sturgeon farms usually breed different species together. The present study reports the first cases of AcIV-E infection in starry sturgeon (*A. stellatus*) and in sterlet (*A. ruthenus*) reared in co-habitation with Russian sturgeon on a sturgeon farm in Northern Italy. Our hypothesis was that passage of the virus from the Russian sturgeon to the other Acipenseridae species would occur, as the farm has a history of AcIV-E positivity. Virological analysis was performed only using molecular methods as it is not yet possible to isolate AcIV-E on cell monolayer [2].

## 2. Materials and Methods

### 2.1. Fish Sampling: Starry Sturgeon

**Figure 1:**
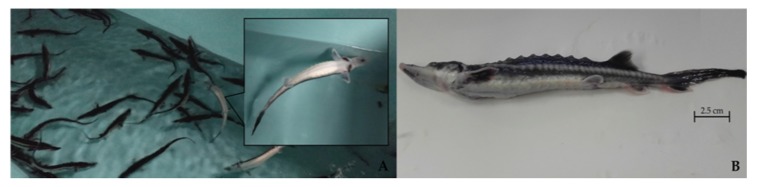
(**A**) Starry sturgeon (*A. stellatus*) reared in a cement tank: The sick fish (**B**) have a swollen abdomen and swimming impairment.

In April 2018, a batch of five starry sturgeon (*A. stellatus*) were analyzed at the Fish Diseases Laboratory, Istituto Zooprofilattico Sperimentale del Piemonte, Liguria e Valle d’Aosta, Turin, Italy. Sturgeon were reared in cement tanks filled with groundwater (temperature range 14.5–15.5 °C) at a density of 20 kg/m^2^. The fish were nine months old, the average length was 25 cm, and the average weight was 200 g. The fish had initially been bred in a hatchery for four months where no clinical signs were observed. They were then moved to other indoor tanks. This batch consisted of 900 starry sturgeons equally divided into two separated tanks. Five months later they began to display uncoordinated swimming and swollen abdomens (Figure 1), which caused them to float. This hindered feeding ultimately led to death by starvation. Cumulative mortality reached approximately 25% of the fish over a period of about 9–10 months. However, not all sturgeons were affected (~15–20%) as the bigger ones appeared healthy. 

In the same farm, there were also several tanks inhabited with AcIV-E positive Russian sturgeons. Farming conditions were like those described above for starry sturgeon. Sturgeons were aged 6–7 months, with an average weight of 70 g. Density of individuals was 25 kg/m^2^. The clinical signs observed were variable depending on the tank considered, with the most frequent signs being hyperactivity and initial lack of food intake. Even mortality rates were extremely variable depending on the tanks (from 25–30% to 90%).

### 2.2. Fish Sampling: Sterlet 

**Figure 2:**
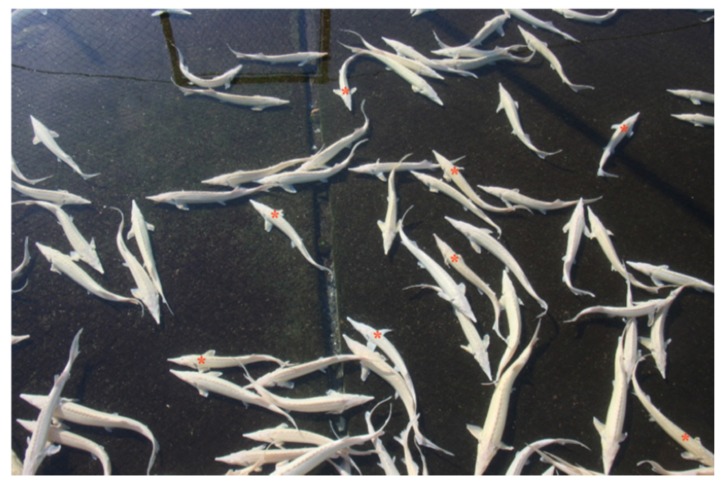
Sterlet (*A. ruthenus*) reared in a raceway system; note the difference in size (red asterisk indicates small individuals).

Five specimens of the albino variety of sterlet (*A. ruthenus*) were sampled from the same farm in December 2018. They had been reared in a raceway system (stocking density of about 30 kg/m^2^). Water supply was provided by groundwater (temperature range 13.5–15.0 °C). The fish were 20 months old (average weight 275 g; range 200–350 g). This sturgeon batch had been imported from a Central European country in April 2017 and reared in the hatchery for four months before transfer to the raceway system. Here, the group consisted of 4800 sterlet farmed together in a single raceway. Anorexia and weight loss were observed, followed by lethargy, and death due to starvation. The percentage of affected fish accounted for a small amount of the total (~10%), as observed in the starry sturgeon. The healthy and the sick fish differed in size, with 200–250 g for the sick and 300–350 g for the healthy (Figure 2). Within a year, about 20% of the sturgeon died. 

During the same period, several batches of Russian sturgeon were farmed and tested positive for AcIV-E. In particular, these sturgeons were bred in the upstream compartments of the raceways system and the starry sturgeon were located in downstream tanks. Farming conditions, age, size, and clinical signs of Russian sturgeons were the same as described in the Section 2.1.

### 2.3. Laboratory Exams

At the laboratory, the fish were euthanized with a lethal dose (200 ppm) of tricaine methanesulfonate (MS-222; Merck, Germany).

For anatomopathological examination, the fish were dissected with clean sterile instruments to prevent contamination with the environment and other fishes. A portion of the gills, which is the target organ for AcIV-E, was removed for virological analysis [9]. Analysis was performed on single and pooled gills for both fish species. 

Parasitological examination involved observation of gill lamellae, cutaneous, and gill mucus at increasing magnification (10x, 20x, 40x) under optical microscopy (Olympus BX40 Clinical Microscope; Microscope Central, USA). The abdomen was dissected and examined for macro- and microscopic intestinal parasites. 

For the bacteriological exam, samples were aseptically taken from the brain and the kidney with a sterile loop, inoculated on Columbia blood agar (Liofilchem, Italy), and incubated at 22 ± 2 °C for 72 h. Colonies were subcultured and subjected to biochemical tests with API 20E (bioMérieux, France) and API 20NE (bioMérieux) for Gram-negative. Identification of isolates was confirmed by Matrix-assisted laser desorption ionization time of flight (MALDI-TOF) mass spectrometry (MS) on a VITEK MS system (bioMérieux). 

### 2.4. DNA Extraction and Real-Time PCR

DNA was extracted from the gills using a QUIamp DNA Mini Kit (Quiagen, Germany) following the manufacturer’s protocol and then stored at −20 °C until use. 

The pair of primers oPVP346 and oPVP347 and the probe tqPVP20 described by Bigarré and coworkers [2] were used to amplify a fragment of the major capsid protein (MCP) of AcIV-E with real-time PCR (qPCR), following the protocol described by the authors. A plasmid with the AcIV-E MPC gene sequence was used as positive control, while nuclease-free water was the negative control for each PCR. DNA from the gills was first tested in the pooled sample, then in single samples to detect the presence of AcIV-E in each fish. Reactions were performed on a StepOnePlus™ real-time PCR System (Applied Biosystems, Foster City, CA, USA).

### 2.5. MPC Gene Sequencing and Phylogenetic Analysis

DNA of positive samples was further amplified with primers oPVP339, oPVP340, oPVP341, and oPVP344 taken, again, from Bigarré and coworkers [2]; PCRs were done on a 2720 Thermal Cycler (Applied Biosystems). Following electrophoresis on 2% agarose gel (Merck), the amplified samples were purified and sequenced according to the Sanger method.

Sequencing results were analyzed using the nucleotide basic local alignment search tool (BLAST) to achieve virus identification. The sequences were aligned using the CLUSTALW algorithm, [10] and a phylogenetic tree was built with MEGA X [11] using a portion of 1240 bp of the MPC gene. The tree was built using AcIV-E sequences previously detected in sturgeon bred or exported from Italy [1,2,7]. The statistical method was maximum likelihood analysis using a General Time Reversible (GTR) model; a bootstrap test was performed using 1000 replications. NV was used as outgroup strain. 

## 3. Results

### 3.1. Starry Sturgeon

**Figure 3:**
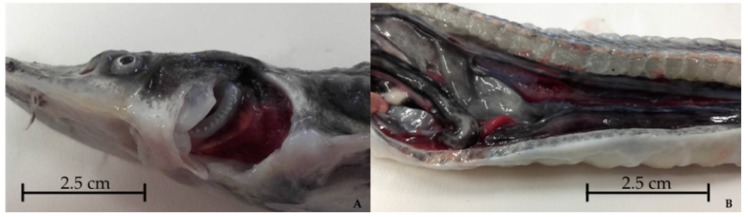
Anatomopathological examination of a starry sturgeon (*A. stellatus*) showing increased gill volume (**A**) and atrophy of internal organs (**B**) in specimen no. 37849/3.

Anatomopathological examination revealed abdominal swelling due to gas accumulation in the stomach and/or intestines, increased gills volume due to edematous change (Figure 3A) and hepatic marbling, as well as atrophy of internal organs (Figure 3B) and splenomegaly. Table 1 presents a detailed list of gross pathology observations. Parasitological and bacteriological exams were negative. 

Biomolecular analysis of the pooled gills detected AcIV-E DNA. qPCR performed on individual gill samples identified AcIV-E DNA in all specimens. Five normal-sized sturgeon were also tested and showed negative results for AcIV-E qPCR. 

### 3.2. Sterlet

**Figure 4:**
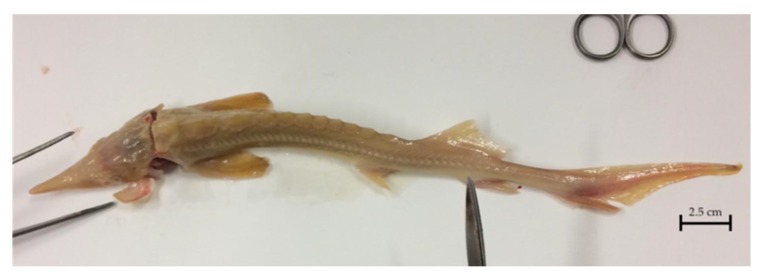
Anatomopathological examination of a lean specimen of sterlet (*Acipenser ruthenus*).

All sturgeon subjected to necropsy were lean (Figure 4) and had congested gills. Table 2 presents a detailed list of gross pathology observations. Hepatic hypoplasia was observed in two fish. The parasitological exam was negative, however the bacteriological exam detected *Shewanella putrefaciens* in one specimen. As in the starry sturgeon, qPCR detected AcIV-E DNA in the gills pool and in all specimens. Five larger size sterlets were tested and didn’t show positivity for biomolecular assays. 

### 3.3. Phylogenetic Analysis

**Figure 5:**
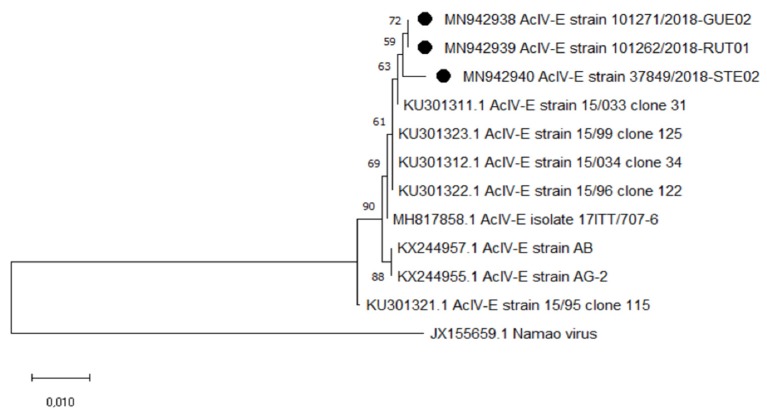
Phylogenetic tree constructed using partial MCP gene sequences (1240 bp) of Italian strains of Acipenser Iridovirus European. Namao virus (NV) was used as outgroup. GenBank accession number of study strains (indicated with “•”): MN942938 (AcIV-E in *A. gueldenstaedtii*), MN942939 (AcIV-E in *A. ruthenus*), and MN942940 (AcIV-E in *A. stellatus*).

The MCP gene sequences were highly similar to virus previously identified in Italy, with nucleotide identities between 99.38% and 99.69%. The phylogenetic tree highlighted the identity of AcIV-E strains detected in the Russian sturgeon (MN942938) and in the sterlet (MN942939). Conversely, there were slight differences between the *A. gueldenstaedtii* strains and the virus identified in the starry sturgeon (MN942940; Figure 5). 

## 4. Discussion and Conclusions

To the best of our knowledge, this is the first report of AcIV-E infection in starry sturgeon and in sterlet. The detection of the virus in a new species of the Acipenseridae family underscores the importance of the disease and its impact, with potential economic and production losses for sturgeon farms. Several aspects still need to be clarified, however. 

First, the disease shows no pathognomonic clinical signs. While it may appear that AcIV-E infection inevitably led to death due to lack of nutrition in both sturgeon species, the conditions differed. In the starry sturgeon we observed uncoordinated swimming and swollen abdomen. Death by starvation and erratic swimming have been reported in previous cases of AcIV-E infection [2,12]. Aimless swimming is also a common characteristic of other viral fish diseases, such as *Acipenserid herpesvirus 2* (AciHV-2) [13] and *Betanodavirus* [14]. In the sterlets, lethargy rather than uncoordinated swimming was observed; again, mortality appeared to be due to starvation. This clinical picture resembles that described by Bigarré and coworkers [2] for Russian sturgeon. A substantial difference in clinical signs described in other species is the difference in size between healthy and sick individuals; none of the previous reports documented such discrepancies. 

Age does not appear to be a factor limiting the onset of virosis; the AcIV-E-positive sterlets (20 months) were twice as old as the starry sturgeon (nine months). Previous reports share this observation, as cases are documented in both juveniles and adults of several sturgeon species (*A. gueldensteaedtii, A. naccarii, A. baerii*) [1,2,7]. 

Anatomopathological examination showed no specific clinical signs, as established on initial inspection. Gill changes were noted in both species; such alterations may be due to a possible epithelial tropism of AcIV-E, as hypothesized by other authors [1]. What remains to be seen is whether the hepatic marbling in the starry sturgeon is a para-physiological condition or a pathological finding.

Given the lack of specific clinical signs and the current impossibility to isolate AcIV-E from fish cellular monolayers [1,2], the only diagnostic means at our disposal is the detection of specific AcIV-E nucleic acid fragments [12]. The choice of primers was based on those used in previous studies [2,7]. In fact, we believe that standardizing the diagnostic process is the best way to increase the little information currently available. Regarding the present study, all specimens of starry sturgeon and sterlet with symptoms tested positive on biomolecular analysis, indicating the presence of AcIV-E DNA. 

Our findings suggest that AcIV-E infection occurred after fish introduction. Since the farm has a history of outbreaks of AcIV-E infection in Russian sturgeon, the infection of starry sturgeon and sterlet probably resulted from transmission of the pathogen from the infected Russian sturgeon. Nonetheless, symptoms and mortality rates differed for the Acipenseridae species.

Phylogenetic analysis supports our hypothesis for the passage of the virus from the infected Russian sturgeon to the other Acipenseridae species. The controls conducted by our laboratory before the importation of the starry sturgeon and sterlet batches documented negativity to AcIV-E. The imported fish began to show clinical signs several months after being introduced to the farm where the infected Russian sturgeon were present. The phylogenetic tree highlights the perfect identity of AcIV-E sequences of *A. gueldenstaedtii* and *A. ruthenus*, while there are differences with the *A. stellatus* strain. Nevertheless, these differences are slight, also compared to the other Italian strains, confirming the results of previous work [2].

AcIV-E infection is one of the main causes of economic loss for European sturgeon farms. The virosis can lead to direct losses due to fish mortality; the main cause seems to be death by starvation. Size discrepancies in sterlets are another cause of economic losses for breeders. AcIV-E infection can also lead to the onset of bacterial diseases caused by opportunistic pathogens or environmental bacteria [1,15]. For example, we isolated *S. putrefaciens* in an AcIV-E-positive sterlet. This ubiquitous rod-shaped Gram-negative bacterium [16] has been isolated in *A. baerii* in a case of co-infection with another pathogen (*Staphyloccocus warneri*) [17]. 

The lack of pathognomonic clinical signs and the impossibility to isolate the virus on cell monolayers remain major limitations in the diagnostic process. Health monitoring should be adopted as a preventive measure for the study and monitoring of AcIV-E infection on sturgeon farms. Control and certification of sturgeon entering the farm are fundamental measures for containing the disease. 

## Figures and Tables

**Table 1:** Specimen number, gross pathology, and parasitological, bacteriological, and molecular analysis of starry sturgeon (+ positive; - negative). C, control; Ct, cycle threshold.

Specimen	Gross Pathology	Parasitological Analysis	Bacteriological Analysis	qPCR (Ct)	End Point PCR
37849/1	abdominal swelling, increased gill volume, fatty liver, atrophy of internal organs	-	-	+ (35.49)	+
37849/2	abdominal swelling, increased gill volume, fatty liver	-	-	+ (33.06)	+
37849/3	abdominal swelling, increased gill volume, fatty liver	--	-	+ (36.92)	+
37849/4	abdominal swelling, increased gill volume, fatty liver, splenomegaly	-	-	+ (32.85)	+
37849/5	abdominal swelling, increased gill volume, fatty liver, splenomegaly	-	-	+ (32.59)	+
C +	//	//	//	+ (18.32)	//
C –	//	//	//	- (no Ct)	//

**Table 2:** Specimen number, gross pathology, and parasitological, bacteriological, and molecular analysis of sterlet sturgeon (+ positive; -negative). C, control; Ct, cycle threshold.

Specimen	Gross Pathology	Parasitological Analysis	Bacteriological Analysis	qPCR (Ct)	End Point PCR
101262/1	thinness, congested gills, hepatic hypoplasia	-	-	+ (25.44)	+
101262/2	thinness, congested gills	-	-	+ (25.73)	+
101262/3	thinness, congested gills, hepatic hypoplasia	--	-	+ (22.76)	+
101262/4	thinness, congested gills	-	-	+ (25.65)	+
101262/5	thinness, congested gills	-	*Shewanella putrefaciens*	+ (23.42)	+
C +	//	//	*//*	+ (16.77)	//
C -	//	//	*//*	- (no Ct)	//
